# Barriers and facilitators to exclusive breastfeeding in rural Pakistan: a qualitative exploratory study

**DOI:** 10.1186/s13006-022-00495-4

**Published:** 2022-08-19

**Authors:** Atif Riaz, Shelina Bhamani, Sheraz Ahmed, Fayaz Umrani, Sadaf Jakhro, Abdul Khaliq Qureshi, Syed Asad Ali

**Affiliations:** 1 Department of Pediatrics and Child Health, Aga Khan University, Karachi, Pakistan; 2 Department of Obstetrics and Gynaecology, Aga Khan University, Karachi, Pakistan

**Keywords:** Barriers, Facilitators, Exclusive breastfeeding, Rural Pakistan

## Abstract

**Background:**

Exclusive breastfeeding (EBF) of children until six months of age is considered one of the most critical interventions in tackling childhood undernutrition. EBF rates are suboptimal in Pakistan, particularly in rural areas where child undernutrition is most prevalent. This study aimed to explore barriers to EBF in a rural context of Pakistan.

**Methods:**

The study was conducted in the rural district Matiari of Sindh, Pakistan, during Jan-March 2020. We used a qualitative exploratory study design and conducted 36 focus group discussions (FGDs). Participants were purposively selected mothers who had not practiced EBF during their previous childbirth, their spouses and mothers-in-law, and lady health workers (LHWs) serving in the study catchment. FGDs were audio-recorded, transcribed, and translated into English from the local language and analysed using thematic content analysis.

**Results:**

Barriers to EBF included low awareness and cultural practices of prelacteal feeds, insufficient breast milk production, undernutrition of mothers, mothers’ occupation as fieldworkers, less birth spacing, low awareness about the correct technique of breastfeeding, maternal and child ailments, abnormal breasts, and influence of in-laws to start top-up feeds. Several facilitators were identified: family support, appropriate maternal diet, maternal awareness, and support in the neighborhood.

**Conclusion:**

Barriers to EBF are multifaceted in rural areas, and interventions aiming to improve adherence to EBFshould be multipronged. Awareness-raising alone might not be sufficient, and other interventions should be designed to address the barriers of maternal malnutrition, insufficient milk production, and socio-cultural practices. In addition, safe alternatives to breast milk may be necessary if breastfeeding is truly not feasible. Lack of breast milk substitutes is particularly challenging for poor rural women who cannot afford infant formula milk.

## Background

Pakistan has one of the largest birth cohorts globally, with over five million children born each year [1]. There has been a slow decline in infant, and child mortality rates over the past decade and currently, infant and under-five child mortality rates are 62 and 74 deaths per 1000 live births, respectively [2, 3]. Malnutrition is considered one of the most important underlying causes of child mortality, with almost 50% of all under-five deaths attributable to undernutrition [4, 5]. A national nutrition survey indicates abysmal conditions for childhood nutrition in Pakistan, with one-third of children being underweight, 40.2% stunted and 17.7% wasted [6]. The situation is even worse in Sindh province, where 41.3% of children are underweight, 45.5% stunted, and 23.3% wasted, with rural areas worst than urban areas [6].

Exclusive breastfeeding (EBF), as recommended by the World Health Organization (WHO), is considered one of the most critical and cost-effective interventions in tackling childhood undernutrition worldwide. It can also decrease by 10% the disease burden in children younger than five years [7]. Furthermore, the WHO recommends early initiation of breastfeeding i.e., within one hour of childbirth, and discourages use of prelacteal feed. Evidence suggests that those neonates who receive breastfeeding after one hour of birth are at higher risk of dying within the first month of life compared to those who are breastfed within one hour of birth (pooled OR 2.02; 95% CI 1.40, 2.93) [8]. Moreover, delayed initiation of breastfeeding is also associated with perceived insufficient milk supply and discontinuation of EBF [9]. However, EBF practice in Pakistan has been sub-optimal. According to the Pakistan Demographic and Health Survey (PDHS), only 48% of children less than six months of age are exclusively breastfed and 53% of children continue to breastfeed until two years of age [3].

Studies to explore context-specific socio-cultural barriers and facilitators are required to develop strategies that are responsive to the local needs. There have been very few studies in Pakistan exploring barriers to EBF. These studies either had a limited number of participants enrolled from hospital settings or focused on working mothers from urban areas [10–13]. Moreover, previous studies in the Sindh province of Pakistan have reported associations based on survey data and did not provide an in-depth understanding of perceptions about EBF from various stakeholders at the household and community levels [14]. A qualitative study from Rajanpur district of Punjab province has reported breastfeeding practices and behavior of parents [15], but did not account for diverse views which can possibly come from mothers-in-law and LHWs since their influence in decision making at household and community level has been documented previously [12, 16, 17]. This study aimed to explore barriers and facilitators to EBF in a rural district of Sindh, Pakistan from the perspective of various stakeholders at the household and community levels.

## Methods

### Study setting

This study was conducted in the Matiari district of Sindh, Pakistan. Around 80% of the population in Matiari lives in rural areas, with a total population of about 0.8 million [18]. There are three Taluka (sub-districts) with a minimum of four and a maximum of eight union councils in each. A union council is the smallest administrative unit in a district with a population of 25,000 – 30,000. A minimum of three union councils were purposively selected from each Taluka to assess child feeding practices and barriers and facilitators to EBF. This study was piggybacked onto ongoing research work in the district (Study of Environmental Enteropathy and Malnutrition in Pakistan (SEEM Pakistan) [19]. The project team had identified mothers who had not breastfed their children exclusively.

### Study design and data collection

A qualitative exploratory research design was used for this study. Data were collected during January to March 2020 using focus group discussions (FGDs). The target population included lactating mothers who had children under two years and did not breastfeed exclusively during the first six months of childbirth; their spouses, and mothers-in-law. Lactating mothers were identified in the parent study through a cross-sectional survey. At least one FGD was conducted with each group of purposively selected lactating mothers, their spouses, and mothers-in-law at selected union council (Table 1). In addition, at least one FGD was conducted with lady health workers (LHWs) of the respective union council since they are advocates for breastfeeding in the community and are aware of the societal dynamics. LHWs are frontline community health workers responsible for visiting the community and counseling mothers to exclusively breastfeed up to six months and to continue breastfeeding with complementary foods until two years [20]. There were 8 – 12 participants in each FGD.

**Table 1:** Focus group participants and areas explored

Type of participants	Number of FGDs	Number of participants	Union councils	Themes explored
Lactating mothers	9	79	Hala-2, Karam Khan Nizamani, Old Hala, Matiari, Shah Alam Shah Ji Wasi, Sekhat, Shah Mir Rahu, Bhalidino Kaka, Zairpir	Perceptions about most appropriate diet for children aged less than six months Importance of breast milk and feeding practices for children aged less than six months Decision making about the first diet of baby Alternatives being used along with breastfeeding Barriers faced for EBF Facilitators for EBF
Spouses	9	90	Hala-2, Karam Khan Nizamani, Old Hala, Matiari, Shah Alam Shah Ji Wasi, Sekhat, Shah Mir Rahu, Bhalidino Kaka, Zairpir	Perceptions about most appropriate diet for children aged less than six months Importance of breast milk and feeding practices for children aged less than six months Decision making about the first diet of baby Barriers faced for EBF Facilitators for EBF Challenges faced to acquire balanced diet for lactating mothers
Mothers-in-law	9	77	Hala-2, Karam Khan Nizamani, Old Hala, Matiari, Shah Alam Shah Ji Wasi, Sekhat, Shah Mir Rahu, Bhalidino Kaka, Zairpir	Perceptions about most appropriate diet for children aged less than six months Importance of breast milk and feeding practices for children aged less than six months Decision making about the first diet of baby Alternatives being used along with breastfeeding Barriers faced for EBF Facilitators for EBF Frequency of LHWs visits and services provided LHWs counseling on EBF
Lady health workers	9	99	Hala-2, Karam Khan Nizamani, Old Hala, Matiari, Shah Alam Shah Ji Wasi, Sekhat, Shah Mir Rahu, Bhalidino Kaka, Zairpir	Perceptions about most appropriate diet for children aged less than six months Importance of breast milk and EBF Challenges for nursing mothers of EBF Facilitators for nursing mothers of EBF

*EBF* exclusive breastfeeding, *FGD* focus group discussions, *LHW* lady health workers

FGDs were conducted by a senior social scientist (moderator) and a research assistant (notetaker). Participants were invited for FGDs at a time and place convenient to them. Participants were asked individually about demographic variables such as age, profession, and educational status before initiating group discussions. Education until matriculation level indicates successful completion of secondary school. The moderator initiated the discussion in the local language using a pre-tested semi-structured guideline which included questions on breastfeeding practices, barriers to EBF, and facilitators to EBF during the first six months after childbirth. The note taker ensured all important points and non-verbal gestures were noted. A free flow of discussion was encouraged and probing questions were used where necessary. Furthermore, a participation diagram was used to ensure all participants responded to the questions. All FGDs were audio-recorded after taking consent from the participants and later transcribed and translated into English for analysis. Rigour was ensured by maintaining reflexive journals and cross-checking notes taken during the FGDs for completeness and consistency. Moreover, data credibility was ensured through member checking and debriefing at the end of each FGD [21].

### Data analysis

Thematic content analysis was carried out using qualitative data analysis software NVivo 12.0 by trained research staff [21]. NVivo software helps maintain a trail of all steps of analysis, ensuring transparency. Initially, the transcripts were reviewed by the primary investigator, and responses for each question were brought together through auto-coding in NVivo. Free codes were developed based on issues identified and data were assigned these codes. Free codes were then merged / grouped to identify emerging themes and sub-themes. Responses and codes were reviewed by primary and co-investigators.

### Ethical considerations

Participation in the FGDs was voluntary, and no means of coercion were used to enhance participation. All participants signed a consent form after their concerns and questions about the study were addressed. The confidentiality of each participant was maintained by giving him / her a unique identifier. The study was approved by the Ethical Review Committee (ERC) of Aga Khan University, Karachi (ERC reference # 2019–1186-3375).

## Results

### Demographic characteristics of participants

Altogether, 79 mothers, 77 mothers-in-law, 90 fathers, and 99 LHWs participated in FGDs. The mean age of mothers was 29 years, and 70% of them were illiterate. Forty-nine per cent of mothers were housewives, and 51% were doing labor work (See Table 2).

**Table 2:** Demographic characteristics

Variables	Mothers *n* = 79	Mothers-in law *n* = 77	Fathers *n* = 90	Lady health workers *n* = 99
Age mean (SD)	29.11 (± 5)	58.16 (± 8.75)	40.01 (± 12.5)	37.67 (± 11.5)
Education *n* (%)				
Illiterate	55 (69.6)	68 (88.3)	18 (20.0)	-
Below matriculation	20 (25.3)	9 (11.7)	28 (31.1)	-
Matriculation	3 (3.7)	-	16 (17.7)	43 (43.4)
Intermediate	1 (1.2)	-	10 (11.1)	24 (24.2)
Graduate and above	-	-	18 (20.0)	17 (17.1)
Occupation / livelihood *n* (%)				
Housewife	39 (49.3)	54 (70.1)	-	-
Laborer	40 (50.7)	23 (29.9)	27 (30.0)	-
Health workers	-	-	-	99 (100)
Farmer	-	-	23 (25.5)	
Teacher	-	-	10 (11.1)	
Driver	-	-	5 (5.5)	
Landlord	-	-	4 (4.4)	
Shopkeeper	-	-	6 (6.6)	
Others	-	-	15 (16.7)	

*SD* standard deviation

The mean age of mothers-in-law was 58 years, 88% of them were illiterate, and 70% were housewives. The mean age of fathers was 40 years; 20% were illiterate, while 31% had received education below matriculation, and the rest were above matriculation. Most fathers were associated with labor work (30%), and farming (25%). The mean age of LHWs was 38 years; 85% of them had achieved an education level of matriculation and above.

### Awareness and practices about breastfeeding

Most of the participants from all focus groups mentioned that breast milk is essential for the health and physical growth of children due to its nutritional properties; however, very few participants explained the benefits of EBF. Moreover, a number of participants reported the use of prelacteal feeds instead of early initiation of breast milk. Most mothers, and mothers-in-law mentioned that they give honey, tea (without milk), molasses and jaggery to the baby immediately after birth. Honey was the most frequently mentioned prelacteal feeds. Mothers stated that it is a tradition to offer such items to newborns, and they do it under the influence of mothers-in-law and elders in the home. Most of the mothers also mentioned that they used mixed feeding within the first six months. Moreover, mothers-in-law were of the view that honey and jaggery are good for a baby’s digestive system and should be given immediately after birth. A few participants also mentioned that honey protects children from fever, cough, and other respiratory ailments. It also helps to clear the mucus from the newborn’s body. One of the participants stated:

“First of all, we give honey to the newborn. Elder women told us that it is good, and baby does not get fever or breathing problems.” *(Mother, FGD #1).*

LHWs also mentioned similar cultural practices about prelacteal feeds; however, they believed that these practices are less common these days. One of the LHWs mentioned:

“They believe that prelacteal feed keeps the baby’s digestion well, and baby can pass stool easily. Also, if baby develops chest congestion, it gets well too, this is their understanding but medical findings did not support this claim.Thus it should not happen.” *(LHW, FGD #2).*

### Barriers to exclusive breastfeeding

#### Insufficient breast milk

A number of barriers and facilitators were reported by FGD participants. Table 3 ranks these based on the most number of responses for a particular barrier and a facilitator. Although most of the mothers reported that they breastfed their children, they could not practice EBF. The majority of mothers mentioned that breast milk was not sufficiently produced, and they had to give other food items such as cow’s milk, goat’s milk, tea without milk, or in a few instances, infant formula milk in addition to breast milk for children under six months of age.

**Table 3:** Barriers and facilitators to exclusive breastfeeding ranked based on number of responses

Barriers to exclusive breastfeeding	•Insufficient breast milk •Undernourishment of mothers •Resuming field work •Short birth interval •Baby positioning •Mother’s or child’s sickness •Influence of grandmothers and family •Substance abuse by mothers
Facilitators to exclusive breastfeeding	•Family support •Maternal health and diet

One of the mothers stated:

“Breast milk is important for a child’s health and growth. It is also important to protect the baby from illnesses. But my milk is not sufficient, and the baby’s tummy does not get filled, and I give goat’s milk in addition to breast milk.” *(Mother, FGD #1).*

Another participant stated:

“When breast milk is less produced, then I go to the doctor for a checkup, and he advises me to use powder (formula) milk.” *(Mother, FGD #3).*

#### Undernourishment of mothers

Undernourishment of mothers was another frequently cited barrier by mothers and fathers. Fathers explained that due to poverty, they were unable to buy a variety of foods necessary for the nourishment of mother and child. They had to rely on the available staple food such as ‘lassi’ (a drink made from yogurt) and bread or rice. Other participants mentioned that when the mother is weak (undernourished), insufficient breast milk is produced and the child’s requirements are not fully met.

#### Resuming fieldwork

Several mothers reported that they go to agricultural fields for their routine work, such as harvesting crops, and yielding vegetables. Due to harsh weather conditions, they leave the children at home. Caretakers at home, such as mothers-in-law, are responsible for feeding the children, due to which they cannot practice EBF.

One of the mothers said:

“I give breast milk to my child before going to the fields, but I cannot take my child along as it is too hot outside. If I return late, my mother-in-law feeds my child with yogurt, khichri (wet rice), or a goat’s milk *(Mother, FGD #3).*

A few mothers-in-law reported that mothers also express breast milk for their children before leaving for work.

“... sometimes she keeps her breast milk in a glass for her baby at home. “ *(Mother-in- law, FGD # 2).*

#### Short birth interval

Participants also mentioned that a brief interval between pregnancies results in less production of milk due to which they are unable to continue breastfeeding. A few mothers also perceived that it is not good for a child to be fed breast milk during pregnancy. As stated:

“They do not do break in between pregnancies and the mother does not get a chance to recover, and continue breastfeeding.” *(Mother-in- law, FGD # 4).*

“Sometimes, the mother gets another pregnancy. The first baby is younger than one year. Then she leaves breastfeeding, that is also one reason.” *(LHW, FGD # 3).*

#### Baby positioning

LHWs also mentioned that new mothers were not aware of the techniques of holding children in the appropriate positions for breastfeeding.

“When it is first child, mothers do not know about the position where the baby can easily suck breast. If the position of a baby was right, the baby would breastfeed properly. Girls at our community usually wed in young age, and they need proper training. We often brief them about breastfeeding, and tell them that now you are a mother, you must breastfeed your child.” *(LHW, FGD # 5).*

#### Mother’s or child’s sickness

Mothers and fathers mentioned that breastfeeding is sometimes discontinued temporarily when either mother or the child becomes sick, or mother is getting treatment for any ailment. In addition, a few mothers reported breast and nipple deformities as reasons for discontinuation of breastfeeding. A few mothers also mentioned that their children could not suck properly. Mothers and mothers-in-law also discussed the role of weather affecting breastfeeding as:

“In the winter season, if I put my hand in cold water, then breast milk gets cold, and the baby’s chest gets affected. In such situations, we avoid breastfeeding.” *(Mother, FGD# 6).*

#### Influence of grandmothers and family

Several mothers verbalized that the influence of grandmothers significantly impacts the decision to continue EBF. They perceived that grandmothers being elderly members of the family, are more experienced and knowledgeable in handling children. They often influence mothers to start top-up feeding, in addition, to breastfeed for children less than six months of age, so that their nutritional requirements are adequately met. Top-up feed mostly constitute animal milk. As stated:

“I have given goat’s milk to my children. My elder son was born through surgery, my breast milk was insufficient, and my mother-in-law asked me to give him goat’s milk. She also asked me to make ‘*kichdi’* (cooked mashed wet rice) for him.” *(Mother, FGD # 7).*

Another mother said:

“Our elders have raised us in that way, and now, we are doing the same.” *(Mother, FGD # 7).*

#### Substance abuse by mothers

A few mothers-in-law also perceived that substance abuse by mothers, such as the use of betels and chewing tobacco, makes mothers weak and consequently affects breastfeedings.

### Facilitators

#### Family support

Participants also mentioned factors that facilitate the continuation of EBF, such as support from family members, including husband and mother-in-law.

“If my mother-in-law tells me that I should give breast milk to the baby, I will do that. If she asks me to give yogurt or goat’s milk, then I give that to my child so that he can grow.” *(Mother, FGD # 6).*

#### Maternal health and diet

Fathers and mothers-in-law mentioned that if the mother is healthy and the maternal diet is appropriate, then she can continue to breastfeed the child.

“When she gets pregnant, she should get more bread, or she should eat at least two breads because now she is one-and-a half person. Also, when the baby is born, she should increase her food in the shape of fruits, milk, and there are other things. Then she will be able to feed the baby.” *(Father, FGD #4).*

A few mothers also mentioned that if there are any nursing mother among the neighbors, she can sometimes feed their children when they go to the fields for work.

“When I go to the fields, I give my baby to a neighbor and she breastfeeds my child in my absence.” *(Mother, FGD # 6).*

## Discussion

Exclusive breastfeeding rates are low in the rural districts of Sindh province [6, 22]. This study highlighted that there are several barriers and facilitators to EBF in this rural setting of Sindh, Pakistan. The identified barriers can be mainly categorized into two groups, such as those which can be addressed through awareness campaigns, and those which need measures beyond awareness-raising.

Though the findings suggest that breast milk is perceived to be essential for child health and growth, the rural populations are not much aware of the benefits of EBF. Previous studies in other contexts have also documented similar findings where awareness regarding the importance of EBF was lacking [23]. Women having higher knowledge of the benefits of EBF, and the hazards of bottle-feeding were more likely to practice EBF [13]. Inappropriate practices such as the use of prelacteal feeds, the influence of in-laws for top-up feeds, and lack of maternal knowledge on breastfeeding techniques have also been reported previously in other studies [23, 24]. These barriers can be addressed through awareness-raising, and lactation support programs in rural communities [25]. However, awareness-raising solely focused on mothers may not be enough. Instead, target audience should include mothers-in-law and other influential members of the families along with lactating mothers [26].

The other types of barriers noted in this study are primarily issues beyond the control of lactating mothers. Insufficient breast milk was a frequently reported barrier. A hospital-based study from the Kasur district of Pakistan also noted a similar finding where insufficient milk was considered to be the most critical barrier [10]. Perceived insufficiency of breast milk has been widely reported as a barrier to EBF in local and international studies [27–30]. Whether it is an actual or a perceived barrier needs further exploration. To our knowledge, there is no study available assessing the quantity of breast milk in the rural context of Pakistan.

Furthermore maternal undernutrition was noted to be another challenge resulting in less milk production. While this makes intuitive sense, there is still controversy in the medical literature regarding the claim that poor nutrition of mothers can result in a low quantity of milk. This barrier has not been cited frequently in the literature, and further studies are needed to assess if improving maternal nutritional status or dietary supplementation can improve the quantity, and quality of breast milk.

Unlike urban areas where working mothers often get leave for child care, the rural women have to go to the fields and they find it difficult to carry their children outside in extreme weather. This finding has not been adequately noted and addressed in the past, and interventions aiming to facilitate EBF should consider these socio-cultural factors. Maternal and child ailments, abnormal breasts or nipples, and sucking difficulties by children are other non-modifiable barriers reported in this study and elsewhere [31–33].

This study also found that maternal awareness and support from family and neighborhood can facilitate the continuation of EBF. Furthermore, a few women also mentioned wet nursing. Although this practice is not common in this setting, future studies can explore the potential of wet nursing in Pakistan. Moreover, an appropriate maternal diet is also highlighted as one of the facilitating factors. Further investigation is required to assess the effect of maternal dietary diversity and supplementation on breast milk and EBF practices.

### Limitations of the study

The lactating mothers enrolled in the study were identified in the larger study and were purposively selected to share their experiences, barriers and facilitators of breasfeeding during the first six months following childbirth. They may not be representative of all breastfeeding mothers in the rural areas of Pakistan; therefore the results might not be generalizable.

## Conclusion

Barriers to EBF are multifaceted in the rural areas of Pakistan, and interventions aiming to improve adherence to EBF ought to be multipronged. Awareness-raising alone might not be sufficient, and future research should identify the potential interventions to address the barriers of maternal malnutrition, insufficient milk production, and socio-cultural practices. In addition, safe alternatives to breast milk need to be explored if breast milk is genuinely not feasible, particularly for poor rural women who cannot afford infant formula milk.

## Data Availability

The datasets used and/or analysed during the current study are available from the corresponding author on reasonable request.

## Abbreviations

CI: Confidence interval
EBF: Exclusive breastfeeding
ERC: Ethical review committee
FGD: Focus group discussion
LHW: Lady health worker
OR: Odds ratio
SD: Standard deviation
WHO: World Health Organization
